# A feasibility study of controlled human infection with intradermal Bacillus Calmette–Guérin (BCG) injection: Pilot BCG controlled human infection model

**DOI:** 10.12688/wellcomeopenres.19811.1

**Published:** 2023-10-02

**Authors:** Emma Carter, Ben Morton, Dima ElSafadi, Kondwani Jambo, Tinashe Kenny-Nyazika, Angela Hyder-Wright, Gift Chiwala, Tarsizio Chikaonda, Anthony E. Chirwa, Jonathan Gonzalez Sanchez, Vincent Yip, Giancarlo Biagini, Shaun H. Pennington, Paula Saunderson, Madlen Farrar, Christopher Myerscough, Andrea M. Collins, Stephen B. Gordon, Daniela M. Ferreira

**Affiliations:** 1 Liverpool School of Tropical Medicine, Liverpool, L3 5QA, UK; 2 Malawi-Liverpool-Wellcome Trust, Queen Elizabeth Central Hospital, College of Medicine, Blantyre 3, Malawi; 3 University of Liverpool, Liverpool, England, L69 3BX, UK; 4 University of Oxford, Oxford, England, UK

**Keywords:** Human challenge, Controlled human infection, Human Infection studies, Tuberculosis, TB, vaccination, BCG, Bacillus Calmette–Guérin

## Abstract

Tuberculosis (TB) caused 1.5 million deaths in 2020, making it the leading infectious killer after COVID-19. Bacille Calmette-Guerin (BCG) is the only licensed vaccine against TB but has sub-optimal efficacy against pulmonary TB and reduced effectiveness in regions close to the equator with high burden. Efforts to find novel vaccines are hampered due to the need for large-scale, prolonged, and costly clinical trials. Controlled human infection models (CHIMs) for TB may be used to accelerate vaccine development by ensuring only the most promising vaccine candidates are selected for phase 3 trials, but it is not currently possible to give participants *Mycobacterium tuberculosis* as a challenge agent.

This study aims to replicate and refine an established BCG CHIM at the Liverpool School of Tropical Medicine. Participants will receive an intradermal injection with licensed BCG vaccine (Statens Serum Institut strain). In phase A, participants will undergo punch biopsy two weeks after administration, paired with minimally invasive methods of skin sampling (skin swab, microbiopsy, skin scrape). BCG detection by classical culture and molecular methods will be compared between these techniques and gold standard punch biopsy. Techniques meeting our pre-defined sensitivity and specificity criteria will be applied in Phase B to longitudinally assess intradermal BCG growth two, seven and fourteen days after administration. We will also measure compartmental immune responses in skin, blood and respiratory mucosa in Phase B.

This feasibility study will transfer and refine an existing and safe model of BCG controlled human infection. Longitudinal BCG quantification has the potential to increase model sensitivity to detect vaccine and therapeutic responses. If successful, we aim to transfer the model to Malawi in future studies, a setting with endemic TB disease, to accelerate development of vaccines and therapeutics relevant for underserved populations who stand to benefit the most.

**Registration:** ISRCTN: ISRCTN94098600 and ClinicalTrials.gov: NCT05820594

## Introduction

In 2020 an estimated 10 million people developed tuberculosis (TB) disease and 1.5 million people died, making TB the second leading infectious killer after COVID-19. Multi-drug resistant tuberculosis (MDR TB) is a public health crisis and health security threat, only one in three people with MDR TB were able to access appropriate treatment in 2020 ^1^. Furthermore, an average of 47% of households affected by TB face catastrophic financial costs ^1^. For these reasons, the WHO End TB strategy highlights the need for an effective vaccine to prevent disease as well as safer, easier and shorter treatment regimens ^2^. Despite this, the only currently available vaccine for prevention of tuberculosis is the 100-year-old Bacille Calmette-Guerin (BCG). BCG is effective in preventing disseminated forms of TB but has limited efficacy against pulmonary tuberculosis, the most common and contagious form of TB. Furthermore, it has reduced effectiveness in regions closer to the equator, where the incidence and burden of TB is highest ^3– 5^. The remains an urgent need to develop effective vaccines to prevent pulmonary tuberculosis in these high burden settings.

Multiple factors complicate the development of novel tuberculosis (TB) vaccines. Vaccine trial design is challenging due to the long period between initial infection and progression to disease and there exists a spectrum of disease between latent, incipient, sub-clinical and active disease of which different vaccines may target different stages ^6^. Furthermore, *Mycobacterium tuberculosis* ( *M.tb*) is not reliably detected in clinical specimens from patients with TB creating difficulties in determining endpoints of vaccine trials, in particular as there are no established immunological correlates of protection ^6^. Therefore, there are currently no alternatives to large, costly randomised controlled trials, conducted over prolonged periods ^7, 8^. Therefore, it is important that only the most promising vaccine candidates are selected for progression to these large phase 3 trials. Controlled human infection models (CHIM) have been used to accelerate vaccine development for various infections including malaria ^9^ and typhoid ^10^. Candidate vaccines which have passed successfully through phase I trials and are in Phase 2b efficacy studies could be included in a TB CHIM, which could be designed to either measure prevention of infection (POI) or immunological endpoints. This would allow vaccine discovery to accelerate in a cost-effective manner. Similarly, a responsive TB CHIM could accelerate the development of new drugs and promote refinement of drug combination regimens.

There are current concerns in using wild type *M.tb* as a human challenge agent given that infection cannot be reliably eradicated, treatment is prolonged with toxicities and there is a risk of relapse or recurrent infection ^11^. BCG has an established safety profile in humans and has been used as a vaccination for over 100 years. BCG contains live attenuated *Mycobacterium bovis* which forms part of the *Mycobacterium tuberculosis* complex and is replication competent but does not usually cause clinical TB disease in immunocompetent individuals. A recent systematic review identified several CHIMs using BCG ^11^. Intradermal injection with BCG was associated with expected side effects following routine vaccination with no serious adverse events (SAEs) reported in any of the identified studies. Disseminated BCG-disease has been reported in infants after routine intradermal vaccination and after intravesical installation (as chemotherapy to prevent recurrences of carcinoma in situ and/or Ta/T1 papillary tumours of the bladder) but this is extremely uncommon, primarily affecting immunocompromised individuals and readily treatable using standard anti-tuberculosis therapy ^12^.

We are working in collaboration with partners at the University of Oxford who have established a CHIM using intradermal BCG and have published several peer reviewed articles ^13– 16^. They found optimal BCG recovery with punch biopsies at 2 weeks post intradermal BCG injection and using three times the standard BCG dose, without additional adverse events ^15^. We propose to replicate and further refine this model at the Liverpool School of Tropical Medicine. The primary end point will be determination of mycobacterial recovery by injection site biopsy at 14 days. Furthermore, we aim to perform serial minimally invasive skin sampling paired with respiratory mucosal sampling to investigate and compare the immune response between these compartments using single cell techniques.

## Protocol

### Objectives and outcome measures

**Overarching aim**

To establish the safety and feasibility of an established BCG controlled human infection model in Liverpool, UK.

**Specific objectives ( Table 1)**

1.   Confirm microbiological and molecular recovery of BCG at the intradermal injection site by gold standard skin punch biopsy (punch biopsy) 14 days after challenge

2.   Confirm safety and tolerability in healthy adult participants

3.   Determine agreement between less invasive skin biopsy techniques (including swabs, skin scrapes and microbiopsy) compared to gold standard punch biopsy on microbiological and molecular recovery of BCG with an aim to employ less invasive methods within a refined protocol in future.

4.   Confirm optimal procedures to assess immune response to BCG intradermal challenge locally (skin tissue), systemically (blood) and in the respiratory mucosa (nasal samples).

**Table 1.** Outcome measures: Objectives and endpoints.

	Objectives	Endpoints
**Primary**	To quantify BCG recovered from the intradermal BCG challenge site 14 days after injection	Culture and PCR quantification of BCG at intradermal challenge site by punch biopsy
**Secondary**	Confirm safety and tolerability of study procedures in participants	Actively (solicited) and passively collected data on adverse events
	Confirm agreement between BCG recovery between punch biopsy and minimally invasive skin biopsy	Pairwise comparison between culture and PCR quantification by punch and micro skin biopsy
	Confirm agreement between BCG recovery between punch biopsy and minimally invasive skin scrape	Pairwise comparison between culture and PCR quantification by punch biopsy and skin scrape
	Longitudinal quantification of BCG recovery from the intradermal BCG challenge site at 2, 7, 14, 21 and 28 days	Culture and PCR quantification of BCG at intradermal challenge site by non-invasive skin swab.
	Confirm laboratory assays for immune response to BCG at intradermal injection site	Skin biopsy cell pellet(s) examined for immune cell differentiation and antigen stimulation
	Confirm laboratory assays for immune response to BCG in systemic circulation	Immune cell activation and functional assays; and cytokine levels in blood
	Measure immune response to BCG injection in respiratory mucosa	Nasal scrape pellet and nasal lining fluid examined for cell differentiation and activation and cytokine expression

**Study hypotheses**

1.   We hypothesise that we will recover the BCG SSI strain by tissue biopsy 14 days after intradermal injection by both classical microbiological and molecular diagnostic techniques.

2.   We hypothesise that quantified BCG recovery (molecular techniques) by minimally invasive skin biopsy will be at least 90% as good as gold standard punch biopsy and will offer a more acceptable method for participants in the future.

### Schedule of events and procedures

**Table 2.** Schedule of visits and procedures. Visit 1 may occur up to four weeks before visit 2. X: event scheduled to occur throughout the study. A: event scheduled to occur only in Phase A. B: event scheduled to occur only in Phase B. *: Timeline is approximate only, as exact timings (± time windows) of visits relate to the actual (not intended) date of the previous visit. **in a selected subset of participants only. Phase A: punch biopsy is the gold standard comparator for BCG recovery ^17^ but is invasive, requiring local anaesthetic and a suture to close the wound. Our aim will be to validate less invasive techniques including minimally invasive skin biopsy ^18^ and a skin scrape ^19^ in the pilot phase (n=10) of the study. Phase B (n=20): we will select the optimal minimally invasive skin technique(s) from phase A and apply these longitudinally during study visits to assess BCG growth kinetics and interrogate immune responses over time. No local anaesthetic (LA) is required for minimally invasive biopsy; LA is known to cause artefactual blunting of humoral immune responses in laboratory assays. Nasal and throat swabs will be used to exclude SARS-CoV-2 infection prior to BCG challenge on day 0 (rapid antigen test) and subsequently to check for co-existent respiratory viral infection using PCR techniques. $ If screen >7 days from BCG. Β If virtual chat. Total blood volume per visit 50ml.

Study visit		1	2	3	4	5	6	7
Study day	Initial consent	Screening	Day 0	Day 2	Day 7	Day 14	Day 21	Day 28
Time windows (days)*				±1	±2	±2	±2	±5
Consent (Written)	x	xβ						
Consent (Verbal)	x	x	x	x	x	x	x	x
Eligibility check	x							
History and examination		x						
Vital Signs		x	x	x	x	x	x	x
Urine β-HCG		x	X$					
Screen for AEs			x	x	x	x	x	x
BCG challenge injection			x					
Skin punch biopsy						A		
Skin micro biopsy ^18^			B	B	B	x	B	B
Skin scrape ^19^			B	B	B	x	B	B
Skin swab			x	x	x	x	x	x
Inspect + photograph site			x	x	x	x	x	x
Stitch removal							X	
Diary card provided			x					
Diary card collected								x
FBC		x						
Coagulation		x						
PBMCs		x			x	x		
Serum			x		x	x		
HIV serology		x						
Blood RNA			x		x	x		
Nasosorption			B	B	B	B	B	B
Nasal wash**			B	B	B	B	B	B
Nasal scrape			B	B	B	B	B	B
Nasal & throat swab		B	B	B	B	B	B	B

**Table 3.** Clinical assessment and STOP criteria.

Clinical history and examination	STOP if unexplained or concerning findings on history or examination
Engagement with research team	STOP if the research team have concerns about participant’s ability to commit to frequent communication and safety checks
Illness during study	STOP if participant develops a medical condition or commences medication while on the study that would meet the exclusion criteria
Full blood count	STOP if Hb <10g/l STOP if total WCC <1.5 ×109/l STOP if total WCC >12 ×109/l STOP if platelets <75 ×109/l
Resting SpO _2_	STOP if < 94%

### Study design

Prospective longitudinal controlled human infection study after intradermal injection with BCG SSI in two phases: Phase A is designed to confirm microbiological recovery and determine agreement between the current gold standard of skin punch biopsy 14 days after injection with minimally invasive biopsy techniques. Phase B is designed to refine and optimise immunological assays to measure local (using optimal minimally invasive techniques), systemic (blood) and respiratory mucosal responses to intradermal BCG injection.

**Phase A**

Ten participants will receive a single intradermal injection to the upper arm of BCG SSI 6–24 × 10 ^5^ colony forming units in line with an optimised and established existing protocol ^15^. The BCG SSI strain has been chosen as the UK licensed vaccine for TB. The dose (3x higher than that used in routine vaccination) has been chosen based on study findings by Minhinnick *et al.* ^15^, who demonstrated good mycobacterial recovery at this concentration with no increase in participant adverse events between the doses. Participants will be followed up longitudinally with active assessment for tolerability; adverse events; and serious adverse events. We will determine BCG SSI recovery from the intradermal injection site 14 days later by classical microbiology and molecular diagnostic techniques using gold standard punch biopsy ^15^ as our primary outcome measure. In parallel, and after local anaesthetic injection, we will obtain paired minimally invasive samples (skin swab, skin scrape ^19^ and microbiopsy ^18^ from the same skin site at 14 days and determine agreement of BCG SSI recovery by classical microbiology and molecular diagnostic techniques with punch biopsy

Parameters for progression from Phase A to Phase B are:

1. Participants able to tolerate study procedures
2. No experimental challenge related SAE or SUSAR detected
3. Confirmation of BCG recovery by classical microbiology or molecular techniques from punch biopsy at day 14 in all 10 participants who received intradermal BCG challenge
4. Confirmation of BCG recovery by classical microbiology or molecular techniques from any of the minimally invasive skin sample technique (swab, scrape and microbiopsy) at day 14 in ≥9 participants who received intradermal BCG challenge.

**Phase B**

Twenty participants will receive a single intradermal injection to the upper arm of BCG SSI 6–24 × 10 ^5^ colony forming units in line with an optimised and established existing protocol ^15^. The focus of this phase is to apply and refine standard operating procedures to assess immune responses to intradermal injection at the local (skin), systemic (blood) and respiratory mucosal (nose) compartmental levels. We will employ a refined sampling technique substituting single punch biopsy for longitudinal minimally invasive skin sample technique(s) (as informed by phase A) to measure BCG growth kinetics and local immune responses. In parallel with local immune responses, we will measure respiratory mucosal responses in the nose to determine if/how intradermal BCG injection induces changes in the target immune compartment (respiratory system).

### Study setting

This is a single centre study will be conducted at LSTM in the Accelerator Research Clinics. This bespoke facility is host to experimental human pneumococcal colonisation work in Liverpool with clinical and laboratory staff expert in delivery of controlled human infection model studies. The facility includes dedicated clinical areas; a pharmacy with access to a Qualified Person (QP); and category two and three microbiology and immunological laboratories.

### Controlled human infection model recruitment in the COVID-19 pandemic

During the COVID-19 pandemic, the LSTM group successfully enrolled more than 1000 participants to the ChAdOx1 nCoV-19 vaccine (Astra-Zeneca) phase III trial ^20^. During this period, we have established standard operating procedures to rigorously maintain participant, staff and community safety when attending the facilities. The study site is COVID-19 secure with systems in place to always ensure distancing and minimal access to communal areas. We have set strict inclusion and exclusion criteria to ensure that our participants are not at increased risk of severe COVID-19 disease, including that participants should have received at least two COVID-vaccines ≥ four weeks before study enrolment. In addition, our target age group of 18–50 has a relatively lower risk of severe COVID-19 disease compared to older populations. If COVID is detected (by rapid antigen testing prior to BCG challenge), participants will be temporarily suspended from the study, remaining under observation.

### Participant identification

**Study participants**

Eligibility assessment will be completed in stages:

•   Screening: study nurse or doctor checks that as part of the consent process the potential participant meets all the general screening criteria ( Table 2).

•   Eligibility is confirmed on the screening and follow-up visits. If any STOP criteria (see Table 3) are met, the participant is excluded from and/or exits the study. Eligibility will be signed off by a medical doctor on the eCRF prior to primary intradermal injection.

### Inclusion criteria

•   Healthy adults aged 18–50 (inclusive)

•   Resident near LSTM (<1hr drive) for the duration of the study period

•   Allows the investigators to discuss the volunteer’s medical history with their GP

•   Females of childbearing potential with a negative urine pregnancy test at screening and willing to practice adequate birth control measures during the study.

•   Fluent spoken English - to ensure a comprehensive understanding of the research project and their proposed involvement

•   Capacity to provide written informed consent

•   Able and willing (in the investigators opinion) to comply with all the study requirements

### Exclusion criteria

Exclusion criteria will be self-reported and/or confirmed from GP questionnaire (GPQ) or medical summary if deemed necessary at research clinician discretion

•   Laboratory evidence at screening of latent M. *tb* infection as indicated by a positive ELISPOT response to ESAT6 or CFP10 antigens ^a^

•   Clinical, radiological, or laboratory evidence of current active TB disease ^b^

•   Previous vaccination with BCG, or any candidate TB vaccine

•   Within the last year had close household contact with an individual with smear positive pulmonary tuberculosis

•   Clinically significant history of skin disorder, allergy, immunodeficiency (including HIV), cancer, cardiovascular disease, respiratory disease, gastrointestinal disease, liver disease, renal disease, endocrine disorder, neurological illness or psychiatric disorder.

•   Current medical issues

○ Acute respiratory tract infection in the four weeks preceding recruitment
○ Any uncontrolled medical or surgical condition at the discretion of the study doctor

•   Maternal

○ Female participants who are pregnant
○ Female participants who are lactating
○ Female participants who intend to become pregnant during the study
○ Female participants who are unable to take contraception measures during the study

•   Smoking

○ Current (defined as ≥5/week) or ex-smoker (cigarettes / cigars / e-cigarette / vaping / smoking of recreational drugs) in the last 6 months.
○ Previous significant smoking history (more than 20 cigarettes per day for 20 years or the equivalent [>20 pack years]).

•   Current alcohol and recreational drug use

○ Regularly drinks ≥3units/day (male) or ≥2units/day (female)
○ Uses recreational drugs
○ Participants may be excluded at the discretion of the research clinician

•   Concurrent oral or systemic steroid medication or the concurrent use of other immunosuppressive agents

•   History of anaphylaxis to vaccination or any allergy likely to be exacerbated by any component of the challenge agent

•   Has received any vaccination within one month of screening visit.

•   Has not completed at least two COVID-19 vaccination doses

•   Any abnormality of screening blood or urine tests that is deemed to be clinically significant or that may compromise the safety of the volunteer in the study ^b^

•   Positive HBsAg, HCV or HIV antibodies

•   Current involvement in another trial that involves regular blood tests or an investigational medicinal product ^c^

•   Use of an investigational medicinal product or non-registered drug, live vaccine, or investigational medical device for four weeks prior to dosing with the study challenge agent

•   Administration of immunoglobulins and/or any blood products within the three months preceding the planned challenge date

•   Participants who meet STOP criteria at the time of screening (see Table 3)

•   Any other issue which, in the opinion of the study staff, may

○ Put the participant or their contacts at risk because of participation in the study,
○ Adversely affect the interpretation of the study results, or
○ Impair the participant’s ability to participate in the study.

a: Participants discovered to have evidence of latent *M. tb* infection as defined by a positive ELISPOT test will be referred for a plain chest x-ray. If there is any evidence of active TB disease either on clinical or radiological grounds, further investigation and treatment will be offered under the supervision of a consultant physician in respiratory medicine or infectious diseases.

b: Participants who are excluded from the study because they have been discovered to have a previously undiagnosed condition thought to require further medical attention will be referred appropriately to their GP or an NHS specialist service for further investigation and treatment.

c: Participants will be excluded from the study if they are concurrently involved in another trial that involves regular blood tests or an investigational medicinal product. To check this, participants will be asked to provide their National Insurance or Passport number (if they are a non-British citizen) and will be registered on a national database of participants in clinical trials ( www.tops.org.uk).

### Temporary exclusion criteria

The following are temporary exclusion criteria to inoculation:

- Current infective illness and/or acute infective illness within 14 days of inoculation if COVID-19 negative
- Positive COVID-19 swab whether symptomatic or asymptomatic within 14 days of inoculation
- Currently isolating following exposure to COVID-19
- Antibiotic use within 28 days of intradermal challenge

**Participants that have been temporarily excluded due to a positive COVID-19 swab, will require a negative lateral flow test prior to subsequent intradermal challenge**

Potential participants who are temporarily excluded at screening or prior to inoculation may be re-screened at a later date to assess inclusion into the study. There is no limit to re-screen a potential participant, however, participants would be re-consented if the time since initial written informed consent is greater than 4 months. Participants who meet any of the temporary exclusion criteria may have their participation delayed until resolution of the temporary exclusion criteria or, if this is not possible, will be not analysed as per protocol. If a participant unexpectedly requires a vaccination during the study period, they will remain in the study and will be considered as a part of an intention-to-treat subgroup for the purposes of analysis.

### Protocol procedures

**Screening and eligibility assessment**

Potential participants will be invited to participate in the study using ethically approved advertisements. The following recruitment methods may be utilised to identify and recruit potential participants:

•   Posters/leaflets/large outdoor banners in public and private areas, including but not limited to universities, libraries, golf clubs, community buildings, public buildings, and workplaces including bus and car adverts (all dependent on permission of managers/employers).

•   The intranet/internet/email lists of local universities, colleges, schools, workplaces

•   Social media including Facebook, Instagram, TikTok and Twitter.

•   Local press, television and radio, aiming to include articles/advertisements about the study/interviews with research staff/participants/research ambassadors (with consent).

•   Participants may be approached who are on our mailing list who have previously expressed interest in volunteering for previous studies and are happy to be contacted about future research.

•   Participants may be approached based on their prior consent to receive generic research communications (for example, Accelerator Research Clinic Volunteer Database, the Consent4Consent database or other database).

•   Open Exeter database: Potential study participants will be identified via National Health Applications and Infrastructure Services (NHAIS) who hold the central NHS patient database (Open Exeter). These databases will identify all persons within the local area who are in the appropriate age range. First contact to potential participants will not be made by the researchers. The initial information about the study will be sent out from this agency to preserve the confidentiality of potential participants. Potentially eligible participants will be sent an invitation, which briefly describes the study. Anyone who is interested in taking part will be able to contact the study team by telephone, text or email. Potential participants will then be sent the PIL.

•   We will advertise through community newsletters.

•   We will post flyers in nearby residential locations and do a flyer mail out in nearby residential locations (including mail drops).

•   We will approach people at public engagement events such as International Clinical Trials Day, World Pneumonia Day and other public engagement events3.

•   Sending invites to the public living in a geographical area from the electoral roll.

•   Social or community groups specific to age and target population.

•   We will ask pharmacies to distribute flyers to people collecting prescriptions or display recruitment materials.

•   University announcements pages, mailing lists their social media and freshers events

•   Large local employers to send the REC approved website text with weblinks and fliers out to their employees via email or through their newsletters.

•   We will approach people at asymptomatic swabbing centres and provide fliers or ask them to register on our Accelerator Research Clinic Volunteer Database

•   Other research teams.

The above is not an exhaustive list and further recruitment methods may be utilised if required. Several of the recruitment methods listed above may not be possible during the COVID-19 pandemic. During our recruitment efforts, we will ensure that we always follow government advice, UKHSA guidelines and COVID-19 secure practices

Interested persons are asked to contact the research team by phone or email for further information. Participants who are interested in research can also contact the study team using our established SMS text service. Potential participants will be sent a copy of the participant information leaflet (PIL) and invited to contact a member of the research team if they are still interested in participating. Provided we have not exceeded our capacity for recruitment, prospective participants will be given an unrestricted amount of time to decide whether to participate or not.

### Informed consent

Potential participants will be invited to discuss the study during a 60-minute appointment (including a 30-minute presentation) that may occur as a group discussion. In that circumstance we will follow the latest UKHSA guidance relating to COVID-19. We may use video communication to facilitate this as a group or individually. Participants will be able to discuss consent one-to-one with a registered healthcare professional (doctor or nurse), who is trained in consent, the trial protocol and GCP, and is delegated as per the delegation log. Individuals will be allowed to discuss the study and ask questions privately (if not answered in the group discussion). Potential participants will be asked how long they would like to consider the information and whether they need more time to come to a decision.

To assess capacity to provide consent, participants will be asked to demonstrate that they have understood by communicating their understanding of the study’s objectives, associated risks and potential benefits. This will include completion of a multiple-choice consent quiz to demonstrate their understanding of the study and to ensure researchers have communicated the study information appropriately. Any incorrect answers will be explained by the researcher, with an option to retake the quiz again.

If individuals agree to participate in the study, the study team are satisfied that they meet the eligibility criteria and the participant has voluntarily decided to take part in the research, they will be invited to provide written informed consent and further clinical appointments will be made. The consent form includes an option for participants to allow the DNA/RNA from these blood samples to be studied.

An additional written consent will be taken for punch biopsy on the day of the procedure, by the health professional performing the procedure.

The participant information leaflet (PIL) and presentation (presentation will be based on PIL) will inform them that they may withdraw from the study at any time and that this will not affect the care they receive within the NHS.

Any queries about possible eligibility will be discussed with the CI. A General Practitioner Questionnaire (GPQ) may be sent for specific information regarding medical history, medications and vaccinations if indicated following the medical history at consent. GP questionnaire/ summary will only be requested if indicated during the eligibility assessment and medical questions at consent. This will be reviewed by the study doctor prior to the vaccination appointment if applicable. A continuous verbal consent approach will be used throughout the study at each visit to confirm they are willing to continue.

As part of recommended practice (MRC tissue and biological samples for use in research) participants will be asked to consent to gift their samples for use in future studies and shared with collaborators internationally. All samples will be anonymised.

### The Over-volunteering Prevention System (TOPS)

Consent will be sought to register participants onto The Over-volunteering Prevention System (TOPS) database to guard against the potential for harm that can result from excessive volunteering in clinical trials. This will be done using the participants National Insurance number or passport number (if not a British citizen).

### Randomisation

Participants will not be randomised within this study.

### Blinding and code breaking

Participants and researchers will not be blinded within this study.

### Description of study interventions and study procedures

**Clinical assessment**

Clinical assessment is conducted to confirm and ensure the participant is generally healthy.

• **Review of medical history:** The initial clinic visit will include a focused clinical history including obtaining information on any concurrent medical conditions, medications (including prescription, over the counter and illicit drug use) and vaccination history. Medical history may be confirmed with the GP records prior to the screen visit if there are any concerns or uncertainty remaining after the initial visit where eligibility has been assessed as indicated by the medical doctor.

• **Clinical examination:** The initial clinic visit will include a focused clinical history and targeted clinical examination including recording and evaluating vital signs including heart rate, blood pressure, temperature, oxygen saturations, auscultation of the lung fields and heart sounds. We will also measure weight and height, calculate BMI and estimate lean body mass.

Should a previously unrecognised abnormality be identified, this will be explained to the individual, and all relevant results will be forwarded to their GP so that appropriate investigations and follow-up can be arranged. Further participation will be determined at the discretion of the study doctor. If the participant has a positive HIV or hepatitis test, the result will be given by a suitably trained medical doctor and the participant will be referred to the local HIV/hepatitis service.

Participants may be re-screened at a later date to determine whether they meet eligibility criteria (e.g. if they have an acute infection on the date of screening that has subsequently resolved). They will be reimbursed for the additional study visit.

**Clinical samples (obtained during study visits)**

The following samples will be obtained during the study:

1. **Urine** – Participants will be asked to collect up to 20ml of urine at screening and vaccination visit for pregnancy testing (female).

2. **Nasopharyngeal and throat swab ± saliva** Nasopharyngeal and throat combined swab will be obtained during screening visits and prior to BCG challenge (using a rapid antigen test). The aim is to determine viral infection and recognise potential asymptomatic COVID-19 cases and protect the study participants and staff. IThe PCR tests will be performed in batch at study completion and will not be used to inform study procedures.

3. **Blood sampling** will be performed by trained, experienced staff. Blood volumes taken at each visit are based on the safety blood screening requirements and planned exploratory immunological investigations. The total clinical blood samples taken at each visit will be 50mls, which is a maximum of 350mls over the study.

4. **Punch biopsy** on the intradermal injection site will be performed according to routine dermatological procedures ^21^. Local anaesthetic injection will be required and one to two sutures to close the biopsy site. This procedure is planned to take place only in phase A (n=10 participants) and will be used as the gold standard comparator for minimally invasive skin sampling techniques. Written consent will be taken on the same day as the procedure. The procedure will be carried out by an appropriately trained doctor or nurse experienced in this procedure.

5. **Minimally invasive skin biopsy** on the intradermal injection site will be performed according to published procedures ^18^. This procedure is well tolerated by participants, does not require local anaesthetic; and does not leave a scar. Longitudinal sampling will be performed in Phase B with five circumferential biopsies around the edge of the intradermal injection site taken at intervals described in Table 1, avoiding previous biopsy sites.

6. **Skin scrape** on the intradermal injection site will be performed using a rhinoprobe (see nasal sampling) curette. Our group has established standardised operating procedures using this curette for nasal sampling ^19^. The procedure is minimally invasive and well tolerated by participants for nasal sampling.

7. **Skin swabs** will be rolled across the intradermal injection site at each subsequent study visit to determine if BCG can be accurately recovered and quantified using this technique. There are conflicting reports in the literature on this technique: Blazevic *et al.* ^22^ demonstrated that paired classical and qPCR microbiological quantification techniques demonstrated significant kinetic association. However, another study was unable to culture BCG by swabbing of the injection site ^13^.

8. **Nasosorption** will be obtained because we expect BCG to stimulate the respiratory mucosa. Sample strips are similar to blotting paper and developed by Hunts Development Ltd (UK). Strips collect concentrated nasal lining fluid before the nasal wash to measure inflammatory responses induced by infection that may be associated with increased colonisation density and acquisition. Concentrated nasal fluid will be used to measure cytokine levels by multiplex bead array. Blotting paper will be held inside the nostril for up to 3 minutes until soaked. These will then be removed and placed in a microcentrifuge tube for storage.

9. **Nasal wash will be obtained in a subset of participants only.** This will be performed using a modified Naclerio method. This is a well-used and validated technique to collect nasal bacterial specimens with which we now have 4 years’ experience. Briefly, 5ml of saline is instilled and held for a few seconds in the nares before being allowed to drip in to a sterile Galli pot; this is usually repeated up to 20ml in total. In the event of NW loss (defined as cough/sneeze/swallow) the procedure may then be repeated to obtain an adequate specimen.

10. **Nasal cells** will be collected after nasal wash using a nanosampling method in which cells are obtained through minimally invasive superficial nasal scrape biopsies (rhinoprobe) and participants can be biopsied multiple times with no significant side effects. Up to 4 samples will be obtained at each nasal sampling visit. If no cells are visible on the rhinoprobe following sampling, the sample can be repeated immediately.

**Symptom diary:** We will request that participants complete a symptoms diary for 14 days after intradermal injection. Participants will be asked to grade any symptoms from 0–4 on a Likert scale. This format has been previously utilised in our experimental human pneumococcal carriage studies. The results will be reviewed at visits D2 and D7. Participants will be advised to ensure the clinical team are contacted with any moderate to severe symptoms as soon as possible. Further details on clinical symptom scores and how these will be assessed are described in a specific section below.

**Challenge visit (B)**

***Preparation and administration of mycobacteria***

*Formulation and supply*

BCG SSI contains live attenuated Danish strain 1331 *Mycobacterium bovis* BCG. It is supplied as a powder and solvent for suspension. One vial of SSI reconstituted in 1mL contains 10 doses of 2–8 × 10 ^5^ CFU (see SmPC). BCG SSI will be supplied to the Accelerator Research Clinic by the Liverpool University Hospitals NHS Foundation Trust who stock the vaccine for clinical use. All movements of the study challenge agents will be documented. Challenge agent accountability, storage, shipment and handling will be in accordance with local SOPs. Drug movement will occur in a refrigerated container to ensure a cold chain of +2 to +8°C.

*Storage*

Dispensed BCG SSI vials are supplied in boxes containing multiple vials and each vial is clearly labelled with the market product (BCG SSI). All challenge agents to be administered will be stored in a safe and locked place with no access by unauthorised personnel. The BCG will be stored between +2 ^o^C and +8°C in a locked fridge. The storage conditions will be under the responsibility of the clinical site. Any temperature deviation outside the range +2 to 8°C will be reported to the CI as soon as detected. Following an exposure to such a temperature deviation, challenge agents will not be used until CI approval has been given.

*Administration*

The correct volume will be drawn up and administered intradermally over the deltoid region of the non-dominant upper arm, according to the site-specific SOP. One vial of SSI reconstituted in 1mL contains 10 doses of 2 – 8 × 10 ^5^ CFU (*taken as 5×10 ^5^ CFU for this calculation). To achieve the specified dose of 6 – 24 × 10 ^5^ CFU we plan to reconstitute in 0.4ml (3 doses 0.1ml doses for administration and an additional dose for post hoc checking of dose administered) This volume will be checked in the laboratory prior to study commencement and may be subject to change in order to achieve the target concentration. Intradermal injection will be standardised in the study by using the FDA marked 0.6mm MicronJet microneedle ( https://www.nanopass.com/). After intradermal injection, participants will stay in the unit for 15 minutes and have a clinical review at this point. During the administration of the challenge agent, resuscitation medicines and equipment will be immediately available for the management of anaphylaxis.

**Safety procedures**

Written and verbal instructions are given to the participant describing potential mild, moderate and severe symptoms and the instances to consult the study team.

Participants will be instructed to monitor the development of any symptoms and report them to the clinical team immediately. Home monitoring of symptoms will include a clear flow chart of the necessary intervention should any symptoms develop (see participant safety information sheet).

**Confirmation and quantification of mycobacterial recovery**

Skin biopsy specimens will be cryopreserved and subsequently processed in batch. Typically, each biopsy specimen will be thawed in a 37°C water bath and transferred to an appropriate tissue homogenising platform ^16^. A total of 100 μL of neat homogenate and 100 μL of a 10 ^-1^ dilution, in sterile PBS, will be plated in triplicate onto Middlebrook 7H11 agar. The BCG SSI vaccine vials will be reconstituted in PBS, and 100 μL of appropriate dilutions will be plated in triplicate as positive controls. Plates will incubated at 37°C for 4 weeks before counting. The remaining biopsy specimen homogenate will be stored at −80° for subsequent DNA extraction.

**DNA extraction**

Biopsy specimen homogenate will be thawed, and BCG DNA from 200 μL of homogenate will be released using standard methods. Typically, a tough microorganism (bead beating) lysing kit will be used. Homogenate will be transferred to a separate tube and 50 μL PBS used to wash remaining homogenate from the beads. A total of 180 μL of ATL buffer and 20 μL of proteinase K (Qiagen) will be added, and the homogenate vortexed and incubated at 56°C for 4 hours. Following this step, extractions will be performed as previously described ^16^.

**Quantitative polymerase chain reaction**

Appropriate primers be used for detection of BCG DNA. These are optimised modifications ^16^ of an early template complementary to regions flanking the BCG deletion sequence, RD1, and amplify a 196–base pair fragment ^23^. Polymerase chain reaction (PCR) analyses will be performed as previously described ^16^. A standard curve will be obtained by extracting BCG DNA from 1:10 serial dilutions of 3 pooled BCG Bulgaria vaccine vials in PBS and correcting for live BCG from the corresponding CFU counts on solid agar.

**Antibody measurement**

Samples of serum and nasosorption will be retained for antibody measurement using standard ELISA assays.

**Cellular responses**

Ex Vivo Interferon γ (IFN-γ) Enzyme-Linked Immunospot (ELISpot) assays will be performed on freshly isolated PBMCs from all participants as documented in Table 1. Responses to purified protein derivative (PPD) from M. tuberculosis will also be assessed. Unstimulated PBMCs will be used as a measure of background IFN-γ production. Results will be reported as spot-forming cells per million PBMCs, calculated by subtracting the mean count of the unstimulated PBMCs from the mean count of triplicate antigen wells and correcting for number of PBMCs in the well.

**Genetic responses**

Samples will be retained for transcriptomic signature feasibility testing. These methods will be essential as the CHIM develops for vaccine testing.

**Baseline and subsequent clinical assessments**

Described in Table 1.

**Subsequent visits**

Described in Table 1.

**Sample handling**

Described section 9.6.

**Early discontinuation/withdrawal of participants**

If a participant withdraws from the study for any reason, another participant will be recruited to replace them.

**End of study**

The clinical phase of the study has ended when the last participant completes their last visit. Recruitment and follow up of participants from initial recruitment activities until last participant finishes their last study visit are planned to be completed within 13 months. The end-of-study is completed when the last laboratory assay is analysed on the last participant sample. At this point all samples will be archived unless transferred to the tissue bank with participant consent for future use.

### Safety reporting

**Definition of serious adverse events**

Our safety reporting terms and definitions are described in Table 4

**Table 4.** Safety definitions. NB: to avoid confusion or misunderstanding of the difference between the terms “serious” and “severe”, the following note of clarification is provided: “Severe” is often used to describe intensity of a specific event, which may be of relatively minor medical significance. “Seriousness” is the regulatory definition supplied above.

Term	Definition
**Adverse Event (AE)**	Any untoward medical occurrence in a participant to whom a medicinal product or challenge agent has been administered, including occurrences, which are not necessarily caused by or related to that product.
**Adverse Reaction (AR)**	An untoward and unintended response in a participant to an investigational medicinal product or challenge agent which is related to any dose administered to that participant. The phrase "response to an investigational medicinal product or challenge agent" means that a causal relationship between a trial medication/challenge agent and an AE is at least a reasonable possibility, i.e. the relationship cannot be ruled out. All cases judged by either the reporting medically qualified professional or the Sponsor as having a reasonable suspected causal relationship to the trial medication/challenge agent qualify as adverse reactions. It is important to note that this is entirely separate to the known side effects for the challenge agent. It is specifically a temporal relationship between administration of challenge agent, the half-life, and the time of the event or any valid alternative aetiology that would explain the event.
**Adverse Event of** **Special Interest ** **(AESI)**	An adverse event of special interest (serious or non-serious) is one of scientific and medical concern specific to the challenge agent, for which ongoing monitoring and rapid communication by the investigator to the Sponsor can be appropriate. Such an event might warrant further investigation in order to characterise and understand it. Depending on the nature of the event, rapid communication by the trial Sponsor to other parties (e.g., regulators, DSMC) might also be warranted.
**Serious Adverse** **Event (SAE)**	A serious adverse event is any untoward medical occurrence that: • Results in death • Is life-threatening • Requires inpatient hospitalisation or prolongation of existing hospitalisation • Results in persistent or significant disability/incapacity • Consists of a congenital anomaly or birth defect Other ‘important medical events’ may also be considered serious if they jeopardise the participant or require an intervention to prevent one of the above consequences. NOTE: The term "life-threatening" in the definition of "serious" refers to an event in which the participant was at risk of death at the time of the event; it does not refer to an event which hypothetically might have caused death if it were more severe.
**Serious Adverse** **Reaction (SAR)**	An adverse event that is both serious and, in the opinion of the reporting Investigator, believed with reasonable probability to be due to one of the trial treatments/challenge agents, based on the information provided.
**Suspected** **Unexpected** **Serious Adverse** **Reactions (SUSAR)**	A serious adverse reaction, the nature and severity of which is not consistent with the information about the challenge agent.

**Adverse events**

Table 5 details the expected adverse events of BCG injection and challenge site biopsy

**Adverse event severity assessment**

Unexpected adverse events will be recorded and these AEs will be graded by severity as follows (where 0 = absent, 1 = mild, 2 = moderate, 3 = severe). Table 6 details this process:

**Causality assessment**

The relationship of each adverse event to inoculation must be determined by a medically qualified individual within the study team according to the following definitions:

0 = No relationship:

•   No temporal relationship to BCG intradermal injection

•   Definite alternative aetiology (clinical, environmental or other intervention), and

•   Does not follow pattern of recognised response to BCG intradermal injection;.

1 = Unlikely relationship:

•   Unlikely temporal relationship to BCG intradermal injection or;

•   Alternate aetiology likely (clinical, environmental or other intervention), and

•   Does not follow pattern of recognised response to BCG intradermal injection

2= Likely relationship:

•   Reasonable temporal relationship to BCG intradermal injection, and

•   Event not readily explained by alternative aetiology (clinical, environmental or other interventions), and

•   Similar pattern of response to that seen to BCG intradermal injection, or

3 = Definite

•   Reasonable temporal relationship to BCG intradermal injection and

•   Event not readily produced by alternative aetiology (clinical, environment, or other interventions), and

•   Known pattern of response to BCG intradermal injection.

The chief investigator (CI), with the consultation of the trial steering committee (TSC), if required, will determine causality of AEs. The greatest degree of causal relationship (definite > probable > possible > unlikely related > not related) determined by either the PI or TSC after their discussions will determine the ultimate classification of the AE. Definite (4), probable (3) and possible (2) are considered to be related. No relationship (0) and unlikely (1) are unrelated.

**Table 5.** Expected adverse events.

Expected adverse events
Pain/tenderness at the injection or biopsy site
Redness at the injection or biopsy site
Swelling at the injection or biopsy site
Warmth at the injection or biopsy site
Scaling/pustules at the injection or biopsy site
Transient ipsilateral axillary lymphadenopathy <1cm diameter
Scar at challenge (and biopsy site)
Headache
Fever (>38.3°C)

**Procedure for recording adverse events**

Participants will be asked to complete diary cards for 14 days after BCG challenge. They will be provided with a thermometer and tape measure to enable daily recording of temperature and local reactions at the injection site.

The diary cards will be reviewed at follow up visits and adverse events recorded in the CRF. Outside the diary card periods, expected local and systemic AEs (listed in Table 5) will be specifically asked about and graded by severity (as detailed in Table 6). Participants will be given the opportunity to report any other new symptoms since the last visit with start and stop dates and any treatments undergone.

All AEs (whether reported by the volunteer or solicited by the Investigator) will be clinically assessed at each visit and recorded in the CRF.

The following information will be recorded: description, date of onset and end date, severity and any treatment or intervention undertaken. The severity of events will be assessed on the following scale: 1 = mild, 2 = moderate, 3 = severe.

An assessment of relatedness to the BCG challenge will then be made by the Investigator.

AEs considered related to the BCG challenge as judged by a medically qualified investigator or the Sponsor will be followed either until resolution, or the event is considered stable.

**Table 6.** Grading of adverse events.

Adverse Event	Grade	Measurement
Pain/tenderness at the injection or biopsy site	0 1 2 3	No pain at all Painful on touch, easily tolerated Painful when limb is moved, interferes with daily activity Severe pain at rest, interferes with daily activity
Redness at the injection or biopsy site	0 1 2 3	0mm 1–50mm 51–100mm >100mm
Swelling at the injection or biopsy site	0 1 2 3	0mm 1–20mm 21–50mm >50mm
Fever (oral)	0 1 2 3	≤37.5°C 37.6 – 38.0°C 38.1 – 38.9°C ≥39.0°C
Other AEs	0 1 2 3	Absence of symptom Awareness of symptom but tolerated; transient or mild discomfort; little or no medical intervention required Mild to moderate discomfort leading to interference with usual activity, some minimal medical intervention/therapy may be required. Significant interference with daily activity; some assistance usually required; medical intervention/therapy required; hospitalisation possible

**Reporting procedures for serious adverse events (SAEs) and suspected unexpected serious adverse reactions (SUSARs)**

Any serious adverse event considered by the CI to be related to the challenge agent and unexpected will be reported to the REC. As the challenge agents are vaccines with Marketing Authorisation, the mechanism for reporting any SAEs to the MHRA is via yellow card.

**Follow up and outcome of adverse events**

Those adverse events which are unexpected and likely to be related to the BCG, whether serious or not, which persist at the end of the study will be followed up by the Investigator until their resolution. Additional follow-up visits may be arranged to enable this. As appropriate, participants with ongoing AEs may be advised to consult their General Practitioner (National Health Service) or the study team will arrange specialist review within the NHS. The outcome of all related non-serious AEs and any SAEs will be assessed as:

- Recovered/resolved
- Recovered with sequelae/resolved with sequelae
- Unknown
- Ongoing at end of study
- Fatal

### Expectedness

Expectedness will be determined according to the Summary of Product Characteristics (SmPC).

### Data and safety monitoring committee (DSMC)

A DSMC is an independent committee which will review safety data throughout the study. All roles and responsibilities of the DSMC will be outlined in detail in the DSMC terms of reference. The specific role of the committee will be:

•   To independently review SAEs and AESIs regardless of relatedness to any of the study procedures throughout the study.

•   To formally review the safety profile and quantified BCG recovery rate

•   To perform unscheduled reviews on request of the study team at a demand and frequency determined by the severity of reported adverse events.

The DSMC will be supplied with a safety report at the end of the study, in the event of an SAE, or if requested at any time by the CI or DSMC members.

The Chair of the DSMC will also be contacted for advice where the CI feels independent advice or review is required.

### Pregnancy

Participants who become pregnant during the trial after the challenge may continue trial procedures, including venepuncture, if appropriate, at the discretion of the CI. Participants who become pregnant before BCG challenge will be withdrawn and will not be challenged. The Investigator will collect pregnancy information on any volunteer who becomes pregnant while participating in this trial. The volunteer will be followed up to determine the outcome of the pregnancy.

### Discontinuation

The study will discontinue in the event that BCG challenge has an unacceptable safety profile

### Statistics and analysis

**Statistical analysis plan**

This is a feasibility study, designed to safely transfer procedures from an established BCG controlled human infection model in Oxford to Liverpool. Experience from our collaborators in Oxford suggests that this sample size is a feasible number to recruit, screen, enrol, and follow up in practical terms, whilst also allowing the determination of any substantial differences in BCG quantification and anti-mycobacterial immunity. The sample size has not been determined with the aim of necessarily achieving statistical significance between these outcomes but to provide pilot data for future interventional trials if feasibility is confirmed.

**Statistical methods**

R statistical software Version 4.3.1 will be used for statistical analysis. Grouped immunological data will be assumed to be non-parametric and summarised by medians and inter-quartile ranges. Between-group comparisons will be made using the Mann-Whitney U test. The Wilcoxon Matched Pairs test will be used to make paired comparisons over time within groups. Pearson’s test of correlation will be used to analyse associations between the primary endpoint and secondary immunological outcomes.

### Data management

**Source data**

Source data are defined as all information in original records and certified copies of original records or clinical findings, observations, or other activities in a clinical trial necessary for the reconstruction and evaluation of the trial. All source data are contained in source documents and electronic case report forms (eCRF).

Source documents are defined as the results of original observations and activities of a clinical investigation. Source documents may include, but are not limited to, study progress notes, e-mail correspondences, computer printouts, laboratory data, and drug accountability records.

**Access to data**

All source data produced in this study will be maintained by the investigators and made available for inspection by the Sponsor’s representatives, the REC, MHRA, and any applicable regulatory authorities.

A Trial Monitoring Plan will be developed by the Sponsor and agreed by the Study Management Group (SMG) and CI based on the trial risk assessment. The frequency of monitoring will be dependent on a documented risk assessment of the trial undertaken by the Sponsor. Monitoring will be performed according to ICH Good Clinical Practice (GCP) by the Sponsor. Following written standard operating procedures, the monitors will verify that the clinical trial is conducted, and data are generated, documented and reported in compliance with the protocol, GCP and the applicable regulatory requirements. The investigational site will provide direct access to all trial related source data/documents, eCRFs and reports for the purpose of monitoring and auditing by the sponsor and inspection by local and regulatory authorities.

### Data recording and record keeping

Participant data including the case report formand safety reports will be anonymised prior to archiving or use outside of the direct research team. A unique identification number will be used to identify each participant.

Study data will be recorded directly into REDcap, an Electronic Data Capture (EDC) system or onto a paper source document for later entry into REDcap if it is not available. This includes safety data, laboratory data and outcome data. Any additional information that needs recording but is not relevant for the CRF (such as signed consent forms etc.) will be recorded on a separate paper source document. All documents will be stored safely and securely in confidential conditions.

The electronic CRF (eCRF) must be completed by designated and trained study personnel. It is the responsibility of the Investigator to ensure the eCRFs are completed and submitted to the Sponsor (or designee) in an accurate and timely manner. The processing of eCRFs will include an audit trail (to include changes made, reason for change, date of change and person making change).

### Quality assurance procedures

**Investigator procedures**

Approved site-specific SOPs will be used at the clinical and laboratory sites.

**Modifications to protocol**

No amendments to this protocol will be made without consultation with, and agreement of, the Sponsor. Any amendments to the trial that appear necessary during the study will be discussed by the chief investigator and sponsor concurrently. If agreement is reached concerning the need for an amendment, it will be produced in writing by the chief investigator and will be made a formal part of the protocol following ethical and regulatory approval. The ethics committee will also be informed of any administrative changes of protocol.

**Protocol deviations**

All deviations from the protocol will be documented in a protocol deviation form and filed in the trial master file.

### Monitoring

Data will be evaluated for compliance with the protocol and accuracy in relation to source documents. The trial will be conducted in accordance with procedures identified in the protocol. Regular monitoring will be performed according to ICH GCP. According to applicable SOPs, the Monitors will verify that the clinical trial is initiated, conducted and completed, and data are generated, documented and reported in compliance with the protocol, GCP and the applicable regulatory requirements.

The Trial Steering Committee will meet quarterly to review the progress of the trial. This committee will comprise of the CI, PI, project manager, regulatory manager and lead investigator. The meeting will provide an update of the trial progress and address any administrative, clinical or laboratory issues.

### Risk assessment

A risk assessment and monitoring plan will be prepared before the study opens and will be reviewed as necessary over the course of the study to reflect significant changes to the protocol or outcomes of monitoring activities. Specific risks are outline below.

**Phlebotomy**

The total amount of blood collected over the study period will be a maximum of 350mL. This amount should not compromise otherwise healthy participants. Risks occasionally associated with venepuncture include pain and bruising at the site of venepuncture, light-headedness, and syncope (rarely).

**Nasal, nasopharyngeal and throat samples**

This may cause some discomfort, eye watering or a small spot of blood. Participants may swallow the saline during nasal wash which may taste salty.

**Challenge with BCG SSI**

Full details are given in the summary of product characteristics (SmPC). In brief:

***Local reaction from intradermal BCG injection***

•   An inflammatory reaction as manifested by redness and swelling is expected to occur at the site of injection, followed by a local lesion that may ulcerate to be moist for up to three weeks. This heals over some weeks to months to leave a small flat scar. It is also possible to develop some swelling (<1cm diameter) of axillary lymph nodes.

•   Uncommonly (less than 1 in 100 people) swelling of axillary lymph nodes to more than 1cm across, or an ulcer that discharges fluid at the injection site may occur.

•   Rare side effects (less than 1 in 1000 people) include inflammation of lymph nodes leading to abscesses and discharge of fluid from the swellings.

***Systemic reactions***

•   Systemic reactions to BCG are rare (less than 1 in 1000 people) and include low-grade fever and headache, and allergic reactions. Disseminated complications of BCG, such as bone infections, have also been reported, but are extremely rare and have usually been reported in immunocompromised, not immunocompetent, individuals.

***Allergic reactions***

•   Allergic reactions from mild to severe may occur in response to any constituent of a medicinal product. Anaphylaxis is extremely rare but can occur.

**Skin biopsy**

There is a small risk that the micro or punch biopsy site may become infected. If this did occur, treatment with antibiotics will be prescribed. The punch biopsy site will heal to form a small scar. Allergic reactions from mild to severe may occur in response to any constituent of the local anaesthetic agent – these are extremely rare.

**Risks associated with the COVID-19 pandemic**

To protect participants, Infection control procedures in line with the latest NHS guidance will be used throughout the study. Participants who test positive for COVID-19 at screen will be temporarily suspended from the study to reduce the risk of onwards transmission. Samples provided up to that point will be retained and participation will be continued after the isolation period as suggested by (PHE) or longer (if participant remains unwell).

If a participant does develop symptoms suggestive of COVID-19 infection they will be advised to follow the latest NHS guidance with regards to self-isolation. A clinical review by medical staff will take place in a designated ‘COVID-19’ zone to clarify whether their symptoms are related to COVID-19 infection or the BCG vaccine. This applies to the post challenge period only. A COVID-19 nasopharyngeal swab will be performed at this visit and all further participant study visits suspended until this clarified. In the event that a study participant becomes acutely unwell with COVID-19 symptoms, they will be advised to seek urgent medical attention via normal routes of healthcare.

**Risk to participant contacts**

Intradermal BCG administration is not associated with risk to participant contacts. Very low levels of BCG can be detected on swabbing the moist surface of the injection site, but human-to-human transmission from contact has never been reported.

**Risk to researchers**

Possible risks to researchers include:

- Needle stick injury during venepuncture or vaccination,
- Biological and chemical hazards within the laboratory,
- Risk of COVID-19 transmission

Experienced staff will carry out procedures that are within their competencies, as delegated by the Chief/Principal Investigator, and which are in accordance with relevant SOPs. All of our clinically facing research staff have received at least two doses of COVID-19 vaccination. Appropriate risk and COSHH assessments are in place for clinical and laboratory procedures. All laboratory work will be conducted in an appropriately rated laboratory in line with health and safety regulations for research with human tissues/infectious agents. Personal protective equipment in line with current UKHSA guidance will be used at all study visits according to the procedures carried out.

### Study monitoring

Regular monitoring will be performed according to the study specific Monitoring Plan. Data will be evaluated for compliance with the protocol and accuracy in relation to source documents as these are defined in the study specific Monitoring Plan. Following written standard operating procedures, the monitors will verify that the clinical study is conducted and data are generated, documented and reported in compliance with the protocol, GCP and the applicable regulatory requirements.

### Study committees

**Study Management Group (SMG)**

Includes scientists, health professionals and investigators who provide ongoing management of the trial. They conduct the study and review recruitment and safety reports weekly.

**Study Steering Committee (SSC)**

Provides oversight on trial design, safety, conduct and evaluation consistent with Good Clinical Practice guidelines in accordance with terms of reference. The committee is comprised of members of the SMG as well as members who are independent to the conduct of the clinical trial (which includes the chair) who have expertise in controlled human infection model studies. The SSC consider recommendations from the Data and Safety Monitoring Committee (DSMC) then advise the SMG and Sponsor. The SSC may make major decisions including to terminate the study, replace an arm of the study or amend the protocol.

**Data and Safety Monitoring Committee (DSMC)**

Safeguard and monitor the interests of the trial participants by assessing the safety and efficacy of interventions and review the protocol and statistical analysis plan according to the DSMC terms of reference. They periodically review safety data to determine patterns and trends of events, or to identify safety issues, which would not be apparent on an individual case basis. They may review unblinded data in the interest of safety. Members are independent to the trial, experienced in this field and the conduct of clinical trials. The SSC and DSMC will be provided with interim safety data and at any time the SMG have any concerns regarding the safety of a participant or the general public. The DMSC will advise the SSC and study investigators on whether there are any ethical or safety reasons why the trial should be changed or not continue. The DSMC will meet as per the terms of reference.

### Protocol deviations

A study related deviation is a departure from the ethically approved study protocol or other study document or process (e.g. consent process or administration of study intervention) or from Good Clinical Practice (GCP) or any applicable regulatory requirements. Any deviations from the protocol will be documented in a protocol deviation form and filed in the study master file.

### Serious breaches

A “serious breach” is defined as breach which is likely to affect to a significant degree the safety or physical or mental integrity of the participants or the scientific value of the trial.

The Sponsor will be notified immediately of any case of a serious breach where the above definition applies. The Sponsor of a clinical trial will notify the regulatory authority (MHRA) and ethics committee in writing of any serious breaches of the conditions and principles of GCP in connection with that trial; or the protocol relating to that trial, as amended from time to time, within 7 days of becoming aware of that breach.

All investigators and trial site staff must comply with the requirements of the Data Protection Act with regards to the collection, storage, processing and disclosure of personal information and will uphold the Act’s core principles. Only authorised members of the clinical research team will have access to any participant personal information and only information of direct relevance to the study will be accessed. All electronic records containing personal information will be stored in a password protected database on a password protected computer in the LSTM. Paper documentation containing personal information will be kept in a locked filing cabinet in a locked room in the LSTM.

Each participant will be assigned a unique non-identifiable study number by a member of the clinical research team at recruitment. Unlinked non-identifiable clinical data will be stored and analysed at the LSTM or collaborating laboratories. The study will comply with the General Data Protection Regulations and the Data Protection Act, which requires data to be anonymised as soon as it is practical to do so. The source document and eCRF will be used as the source data for this study. On completion of the study, the eCRF will be locked and source documents will be photocopied and archived in paper and electronically on a secure database. Data will be stored for a minimum of 25 years. The Prof Daniela Ferreira is the data custodian.

The study staff will ensure that participants’ anonymity is maintained. Study participants will be identified a participant ID number on the source document and eCRF. Any electronic databases and documents with participant identifying details will be stored securely and will only be accessible by study staff and authorised personnel. The study will comply with the Data Protection Act, which requires data to be anonymised as soon as it is practical to do so.

### Ethical and regulatory considerations

**Declaration of Helsinki**

The Investigator will ensure that this study is conducted in accordance with the principles of the Declaration of Helsinki.

**Guidelines for good clinical practice**

The Chief Investigator will ensure that this study is conducted in accordance with relevant regulations and with Good Clinical Practice. This trial is subject to approval from the Sponsor and Research and Development at LSTM in addition to the Medicines Healthcare products Regulatory Agency (MHRA) and the National Research Ethics Service (NRES) /Health Research Authority (HRA). 

**Approvals**

Liverpool School of Tropical Medicine are the sponsor for this study, sponsor approval obtained on 23 ^rd^ March 2023.

Ethical approvals for this study have been granted by the Liverpool Central Research Ethics committee (REC reference 22/NW/0373) on 18 ^th^ January 2023.

A substantial amendment (Substantial amendment 1) was submitted and approved by the Research Ethics Committee on 15 ^th^ May 2023.

Substantial amendments that require review by NRES will not be implemented until the REC grants a favourable opinion.

**Other ethical considerations**

We will not seek to recruit vulnerable participants, or participants who are unable to consent for themselves during this study.

### Reporting

All correspondence with the REC will be retained in the Trial Master File/Investigator Site File. An annual progress report (APR) will be submitted by the Chief Investigator to the REC within 30 days of the anniversary date on which the favourable opinion was given, and annually until the trial is declared ended. Within one year after the end of the trial, the Chief Investigator will submit a final report with the results, including any publications/abstracts, to the REC. If the trial in ended prematurely, the Chief Investigator will notify the REC, including the reasons for the premature termination.

The protocol, informed consent form, participant information sheet and any proposed advertising material will be submitted to an appropriate Research Ethics Committee (REC), HRA (where required), regulatory authorities (MHRA in the UK), and host institution(s) for written approval. The Investigator will submit and, where necessary, obtain approval from the above parties for all substantial amendments to the original approved documents.

The design and conduct of the trial will include the principles of autonomy, non-maleficence, beneficence and justice. Participants may learn about clinical research from their experience and there is a possibility of detecting medical problems during clinical examination and their GP may be informed for further investigation as needed. The research is open to all individuals, but important exclusion criteria are in place, primarily to protect individuals from undue risk. This study offers the potential for both local (UK) and global impact in the development of future vaccines and therapeutics as part of the global effort to combat tuberculosis.

### Transparency in research

Prior to the recruitment of the first participant, the study will have been registered on a publicly accessible database.

Where the study has been registered on public platforms, this information will be kept up to date during the study, and the CI or their delegate will upload results to all those public registries within 12 months of the end of the trial declaration.

### Participant confidentiality

The study will comply with the General Data Protection Regulation (GDPR) and Data Protection Act 2018, which require data to be de-identified as soon as it is practical to do so. The processing of the personal data of participants will be minimised by making use of a unique participant study number only on all study documents and any electronic database(s), with the exception of the CRF, where participant initials may be added. All documents will be stored securely and only accessible by study staff and authorised personnel. The study staff will safeguard the privacy of participants’ personal data.

### Expenses and benefits

It is not intended that financial factors influence an individual's decision to participate in this study. The fees will reflect remuneration and not financial coercion. We compensate participants for time, travel, inconvenience and discomfort. The sums offered in this study have been developed over the course of many years for pneumonococcal controlled human infection studies and are consistent with remuneration in other similar local and national studies and are detailed below ( Table 7). 

**Table 7.** Payment summary. The screen and vaccination appointment can be performed as a separate visit or combined as required, the remuneration will be unchanged. Participants will be provided remuneration by direct bank transfer following attendance at visit 7. Remuneration is pro-rata based on the number of visits attended and samples taken. If a participant withdraws from the study early, they will be remunerated for the visits they attended and samples which were taken up until the time they withdrew. If additional visits are required, the participant will be reimbursed for that visit.

Phase A								
	Visit 1	Visit 2	Visit 3	Visit 4	Visit 5	Visit 6	Visit 7	
Samples taken	Screening	Day 0	Day 2	Day 7	Day 14	Day 21	Day 28	
Urine	X	X						
BCG Challenge		X						
Skin punch biopsy					X			
Skin micro biopsy					X			
Skin scrape					X			
Skin swab		X	X	X	X	X	X	
Bloods	X	X	X	X	X	X	X	
Nasosorption								
Nasal cells								
Nose and throat swab	X	X	X	X	X	X	X	
Time taken	60	60	20	20	90	20	20	TOTAL
Remuneration	£40	£50	£20	£20	£150	£20	£20	£320
Phase B								
	Visit 1	Visit 2	Visit 3	Visit 4	Visit 5	Visit 6	Visit 7	
Samples taken	Screening	Day 0	Day 2	Day 7	Day 14	Day 21	Day 28	
Urine	X	X						
BCG Challenge		X						
Skin punch biopsy								
Skin micro biopsy			X	X	X	X	X	
Skin scrape					X			
Skin swab		X	X	X	X	X	X	
Bloods	X	X	X	X	X	X	X	
Nasosorption	X	X	X	X	X	X	X	
Nasal cells	X	X	X	X	X	X	X	
Nose and throat swab	X	X	X	X	X	X	X	
Time taken	60	60	60	60	90	60	60	TOTAL
Remuneration	£40	£55	£55	£55	£55	£55	£55	£370

### Finance and insurance

**Funding**

This project is funded as part of the UKRI Infection Innovation Consortium (iiCON) award: https://www.infectioninnovation.com/

**Insurance**

LSTM has a specialist insurance policy in place which would operate in the event of any participant suffering harm as a result of their involvement in the research (Newline Underwriting Management Ltd, at Lloyd’s of London). NHS indemnity operates in respect of the clinical treatment that is provided.

### Contractual arrangements

Appropriate contractual arrangements will be put in place with all third parties.

### Publication policy

A publication is defined as any written document intended for submission to a congress, conference, journal or other public forum, and includes abstracts, posters and full articles, pertaining to the study, with or without study results. Publications will be consistent with the Consort Guidelines and checklist http://www.consort-statement.org/ and will be based on the International Committee of Medical Journal Editors (ICJME) requirements in that all persons listed as authors must meet ICJME requirements and all persons that meet these requirements must be listed as an author.

The findings from this study will be disseminated amongst the scientific community. We intend to publish our findings in peer reviewed scientific journals and present data at appropriate local, national and international conferences. In addition, we will produce a lay report of our findings, which will be made available to all participants.

**Authorship**

Authorship of the final trial report and subsequent publications will include those who contribute to the design, delivery and analysis of the trial in the study teams and collaborators. Authorship will be defined on completion of the trial in discussion with Professors Stephen Gordon and Daniela Ferreira.

### Development of a new product/process or the generation of intellectual property

Ownership of IP generated by employees of the LSTM vests in LSTM. The protection and exploitation of any new IP is managed by the IP and Research Contracts Team at LSTM.

### Archiving

Archiving will be authorized by the Sponsor following submission of the end of study report and the Sponsor will be responsible for archiving all trial documents and trial databases. All essential documents will be archived for a minimum of 25 years after completion of trial and no study records will be destroyed without prior authorisation from the Sponsor and the data custodian (Prof Daniela Ferreira). Archives will be located at the LSTM.

## Study status

At the time of submission for publication, the study has not commenced recruitment.

## Discussion

The establishment of a safe and effective CHIM for Tuberculosis would accelerate much needed vaccine and therapeutic development. A skin challenge model allows straightforward and reliable isolation of BCG, the key outcome measure of a TB human challenge model. Furthermore, participant procedures are safe and well tolerated and BCG has a long-established safety profile. For this reason, an intradermal BCG challenge model remains the most feasible TB CHIM currently and continued development and refinement of protocols remain important ^11^.

A previously conducted BCG intradermal model found optimal BCG recovery when using three times the dose of the UK licensed BCG SSI ^15^. This model reliably isolated BCG by culture and PCR from punch biopsies and detected a vaccine effect of prior BCG ^13^. We therefore aim to replicate and further refine this model. Given punch biopsy is an invasive procedure, serial sampling with this technique in individual participants is not feasible. Our study is designed to compare BCG isolation for different minimally invasive skin sampling techniques, that may then be used for serial monitoring of mycobacterial recovery and of the compartmental immune response over time, optimising end points of the challenge model for use in vaccine evaluation. 

A recently conducted systematic review and workshop in Liverpool and Malawi highlighted the limitations of an intradermal BCG CHIM in TB vaccine evaluation ^11^. Firstly, given the known limitations of BCG as a TB vaccine, it may not be a reliable surrogate for wild type *M.tb* as a challenge agent. Furthermore, a skin challenge model cannot be used to prove protection against pulmonary infection. Nevertheless, the systematic review and workshop, highlighted this as the most practicable and ethically acceptable model currently. Furthermore, refinement of clinical and laboratory protocols for a TB skin challenge model will facilitate rapid adaptation for novel TB challenge agents that are in development ^24^.

Finally, it is vital that any TB challenge models are developed in collaboration with and conducted in settings endemic for TB, ensuring they are undertaken in relevant populations, given the known reduction in BCG vaccine effectiveness in these regions ^11^. LSTM has established technology transfer processes with previous successful transfer of the experimental human pneumococcal carriage model from Liverpool to Blantyre, Malawi We are therefore working in collaboration with the Malawi Liverpool Wellcome Trust to develop safe and effective procedures and protocols in Liverpool that may then be transferred to Malawi. 

## Data Availability

No data are associated with this article.
