# Supplementary material for: A feasibility study of controlled human infection with intradermal Bacillus Calmette–Guérin (BCG) injection: Pilot BCG controlled human infection model
Source: Wellcome Open Res. 2024 Jun 5;8:424. Originally published 2023 Oct 2. [Version 2] doi: 10.12688/wellcomeopenres.19811.2 (PMC11362739; doi:10.12688/wellcomeopenres.19811.2)
Supplement: Peer review history

# Reviewer report: Reviewer response for version 2

**DOI:** 10.21956/wellcomeopenres.23582.r97056

**Published:** 2024-09-25

**Authors:** Hazel Dockrell

**Affiliations:** 1 Department of Infection Biology, London School of Hygiene & Tropical Medicine, Keppel Street, London, UK

- BCG CHIM models have the potential to provide very valuable information about the immune response to BCG vaccination and mycobacterial infection. To establish a BCG CHIM model in Liverpool and then transfer it to Malawi will be very useful and help us learn more about how BCG vaccination does or does not induce protective immunity to *M.tuberculosis*, including whether the immune responses induced by BCG in the two settings are the same or different.
- The ability to use minimally invasive biopsies or micro biopsies has the potential to enable individuals to be samples over time, to follow the local immune response induced by BCG; if sufficient material can be obtained from these minimally invasive biopsies without the need for punch biopsies with greater scarring, this would be a useful advance.
- The BCG CHIM work proposed has other novel aspects. These include the testing of samples from the nasal mucosa. Giving BCG id should induce a broad immune response including cells that could traffic to the lungs or the nasal mucosa if required in response to antigenic challenge. It will be of interest to see whether local immune responses are detectably altered in the upper respiratory tract without a local antigenic challenge.
- BCG Danish vaccine will be used as the challenge organism. As there can be significant variations in the number of live bacilli, growth rates and in the proportion of live and dead bacilli in different batches of BCG vaccine vials, a single batch of vaccine should be used if possible, and it would be useful to document what the BCG CFU is per vial. For the quantitative PCR experiments, it might be better if BCG Danish could also be used to generate standard curves rather than BCG Bulgaria, which is a different BCG strain.
- The protocol for the two stages of the proposed work has already been reviewed and modified. In the future, it would be valuable to include those with prior BCG vaccination in the UK as well as in Malawi to enable geographic comparisons. Excluding those with cross-reactive responses to PPD is probably not realistic, as many individuals can show such responses due to environmental exposure to mycobacteria etc.
- A minor point is that while BCG may lead to a disseminated BCGosis in a few individuals with an immunocompromised immune system, it will not lead to clinical tuberculosis or TB disease.
- In Table 2, the β symbol should be explained in the legend, is this “B, if virtual chat”?
- Reference 24 describes PCR detection of BCG rather than the development of fluorescent BCG; there is a recent reference for development of fluorescent strains of BCG that might be detected without invasive sampling which could be referenced instead (Krishnan et al PLoS Biol 2024, 22;e3002766).

Is the study design appropriate for the research question?

Yes

Is the rationale for, and objectives of, the study clearly described?

Yes

Are sufficient details of the methods provided to allow replication by others?

Yes

Are the datasets clearly presented in a useable and accessible format?

Not applicable

Reviewer Expertise:

Immunologist, BCG, correlates of protection

I confirm that I have read this submission and believe that I have an appropriate level of expertise to confirm that it is of an acceptable scientific standard.

# Reviewer report: Reviewer response for version 2

**DOI:** 10.21956/wellcomeopenres.23582.r85854

**Published:** 2024-07-01

**Authors:** Mangalakumari Jeyanathan

**Affiliations:** 1 McMaster University, Hamilton, Ontario, Canada

I have reviewed the author's responses to my concerns and found that they have been adequately addressed, with the necessary changes made to the project proposal. I believe the proposal, in its current format, is acceptable for indexing.

Is the study design appropriate for the research question?

Yes

Is the rationale for, and objectives of, the study clearly described?

Yes

Are sufficient details of the methods provided to allow replication by others?

No

Are the datasets clearly presented in a useable and accessible format?

Not applicable

Reviewer Expertise:

NA

I confirm that I have read this submission and believe that I have an appropriate level of expertise to confirm that it is of an acceptable scientific standard.

# Reviewer report: Reviewer response for version 1

**DOI:** 10.21956/wellcomeopenres.21943.r73139

**Published:** 2024-08-29

**Authors:** Paola Villanueva

**Affiliations:** 1 The University of Melbourne, Melbourne, Victoria, Australia

This protocol describes a pilot study of a BCG controlled human infection model (CHIM) (as a proxy to TB model) at the Liverpool School of Tropical Medicine, by replication and refinement of an established BCG CHIM. Two phases are proposed: Phase A to confirm microbiological recovery 14 days after BCG injection, and to compare skin punch biopsy with minimally invasive biopsy techniques. Phase B aims to refine and optimise immunological assays to measure local, systemic and respiratory mucosal responses to BCG injection.

 The study protocol is detailed and contains clinically important participant inclusion and exclusion criteria and appropriate safety measures, relevant to intradermal BCG injection administration.

1. The protocol states that 10 participants will be included in Phase A, and 20 in Phase B. Presumably these will be two different groups of participants, however it would be useful to clearly state this.
2. Is there a contingency plan for Phase B, if there is no ‘BCG recovery by classical microbiological or molecular techniques from any of the minimally invasive skin sample technique at day 14 in at least 9 participants’ (Phase A)?
3. Consideration should be given that repeated skin biopsies at multiple time points may affect the immune response elicited.

Whilst a single BCG SSI strain will be used for this pilot study, it would be interesting to consider comparing with a different BCG strain in the future (such as BCG-Japan), as differences in immunogenicity and efficacy have been reported depending on BCG strain.

Is the study design appropriate for the research question?

Yes

Is the rationale for, and objectives of, the study clearly described?

Yes

Are sufficient details of the methods provided to allow replication by others?

Partly

Are the datasets clearly presented in a useable and accessible format?

Not applicable

Reviewer Expertise:

BCG vaccination safety, tuberculosis diagnostics, antimicrobial stewardship

I confirm that I have read this submission and believe that I have an appropriate level of expertise to confirm that it is of an acceptable scientific standard, however I have significant reservations, as outlined above.

# Reviewer report: Reviewer response for version 1

**DOI:** 10.21956/wellcomeopenres.21943.r73138

**Published:** 2024-02-19

**Authors:** Mangalakumari Jeyanathan

**Affiliations:** 1 McMaster University, Hamilton, Ontario, Canada

The study aims to replicate and improve an established BCG CHIM by identifying a minimally invasive sampling technique that offers a more acceptable approach for participants in the future. To refine the current BCG recovery approach using punch biopsy from the injection site 14 days post-intradermal injection, the proposal suggests comparing minimally invasive skin sampling methods to the gold standard punch biopsy in Phase A. Both microbiological and molecular biological methods will be assessed for BCG recovery. The minimally invasive method that meets the predefined expectation criteria in Phase A will then be utilized to investigate BCG growth kinetics and local and systemic immune responses in Phase B.

 While it is critical to find a convenient sampling method to use in BCG intradermal injection CHIM, I have the following suggestions and concerns.

 It is proposed that for BCG recovery, biopsies will undergo traditional culture for colony-forming units and quantification of BCG DNA. However, detecting DNA in samples does not necessarily reflect live bacterial counts since DNA can persist in the environment even after bacteria have died. While DNA detection can offer insights into the presence of bacterial genetic material, it may not accurately reflect live bacterial counts. Traditional culture methods are reliable for detecting live BCG in samples, but their sensitivity is lower compared to the MGIT assay. The MGIT assay offers faster growth detection and utilizes a liquid medium, increasing the likelihood of detecting BCG even in low numbers. Given the minimally invasive techniques being evaluated, including the MGIT assay will enhance BCG detection.  

 In Phase B, the proposal involves taking skin samples from the injection site at multiple time points after intradermal BCG injection. However, I have concerns about the potential impact of this approach on BCG recovery. Conducting multiple skin biopsies, particularly during the early time points after intradermal injection, may disrupt the localized BCG inoculum, thereby reducing the amount of viable BCG present at the site. This disruption could potentially affect the induction of immune responses and subsequently alter the kinetics of the immune response. Additionally, repeated trauma from the skin biopsies may lead to alterations in the local inflammatory environment at the injection site, potentially due to the recruitment of innate cells. This raises safety concerns and may lead to the formation of granulomas and the persistence of BCG.

 Overall, while multiple skin biopsies at the site of BCG injection may offer valuable insights into the local immune response and BCG dynamics, they also have the potential to affect BCG recovery and subsequent immune responses. Therefore, careful consideration of these factors is crucial when involving repeated biopsies. Additionally, the extensive immune analysis suggested in the study protocol is valuable, particularly if CHIM is compared between healthy controls and individuals vaccinated with BCG or those latently infected.

 If the hypothesis proposed by the authors cannot be met in Phase A, Phase B will need to be redesigned accordingly. The assertion made by the authors that minimally invasive skin biopsies will be at least 90% as effective as standard punch biopsies lacks empirical evidence or justification. Authors should propose an alternative plan if Phase A cannot meet the predefined targets.  

 Given that prior exposure to environmental mycobacteria can influence the outcome of CHIM through cross-reactive immune responses, tuberculin (PPD) skin positivity should be regarded as one of the exclusion criteria. Cross-reactive immune responses can potentially complicate the assessment of BCG recovery. This altered baseline immune status might affect the kinetics and magnitude of immune responses and clearance of BCG. Including participants with pre-existing immune responses to environmental bacteria could significantly impact the interpretation of study outcomes.

Is the study design appropriate for the research question?

Yes

Is the rationale for, and objectives of, the study clearly described?

Yes

Are sufficient details of the methods provided to allow replication by others?

No

Are the datasets clearly presented in a useable and accessible format?

Not applicable

Reviewer Expertise:

Tuberculosis, immune response to vaccines, clinical trial protocol

I confirm that I have read this submission and believe that I have an appropriate level of expertise to confirm that it is of an acceptable scientific standard, however I have significant reservations, as outlined above.

# Response

**Published:** 2024-04-29

**Authors:** Emma Carter

**Affiliations:** Department of Clinical Sciences, Liverpool School of Tropical Medicine, Liverpool, UK

Response to reviewer 2 comments -

 (I) **Reviewer Concern:** It is proposed that for BCG recovery, biopsies will undergo traditional culture for colony-forming units and quantification of BCG DNA. However, detecting DNA in samples does not necessarily reflect live bacterial counts since DNA can persist in the environment even after bacteria have died. While DNA detection can offer insights into the presence of bacterial genetic material, it may not accurately reflect live bacterial counts. Traditional culture methods are reliable for detecting live BCG in samples, but their sensitivity is lower compared to the MGIT assay. The MGIT assay offers faster growth detection and utilizes a liquid medium, increasing the likelihood of detecting BCG even in low numbers. Given the minimally invasive techniques being evaluated, including the MGIT assay will enhance BCG detection. 

**Author Response:** Thank you for this feedback. Since submission of the protocol, the Research Ethics Committee have approved a substantial amendment and we will now perform punch biopsies on day 14 for traditional culture in phase B. This will allow us to correlate PCR results from the serial microbiopsy specimens with the traditional culture at day 14. We will be storing the samples and processing these in batch so will have the opportunity to undertake MGIT culture on these microbiopsy samples if funding is secured for this additional procedure. The protocol has been edited in table 1, Schedule of events and section 9.5.2 Clinical samples to reflect this change.

 ------------------------------------------------------------------------------------------------------------------

 (II) **Reviewer Concern: **In Phase B, the proposal involves taking skin samples from the injection site at multiple time points after intradermal BCG injection. However, I have concerns about the potential impact of this approach on BCG recovery. Conducting multiple skin biopsies, particularly during the early time points after intradermal injection, may disrupt the localized BCG inoculum, thereby reducing the amount of viable BCG present at the site. This disruption could potentially affect the induction of immune responses and subsequently alter the kinetics of the immune response. Additionally, repeated trauma from the skin biopsies may lead to alterations in the local inflammatory environment at the injection site, potentially due to the recruitment of innate cells. This raises safety concerns and may lead to the formation of granulomas and the persistence of BCG.

 Overall, while multiple skin biopsies at the site of BCG injection may offer valuable insights into the local immune response and BCG dynamics, they also have the potential to affect BCG recovery and subsequent immune responses. Therefore, careful consideration of these factors is crucial when involving repeated biopsies. Additionally, the extensive immune analysis suggested in the study protocol is valuable, particularly if CHIM is compared between healthy controls and individuals vaccinated with BCG or those latently infected.

**Author Response:** We have submitted a substantial amendment and will now perform skin samples at fewer intervals (days 2, 7 and 14), with punch biopsy at day 14. Punch biopsy will ensure removal of previously microbiopsied tissue at day 14, minimising the possibility of trauma causing granuloma formation or persistence of BCG. BCG has a long-established safety profile and granuloma formation is rare. Given we are now performing punch biopsy on day 14, BCG recovery can be compared with samples from phase A of the study where serial skin sampling was not performed to assess the effect of serial skin sampling on mycobacterial recovery. We acknowledge that it is a potential limitation of the study that serial microbiopsy may affect the local immune response, we will attempt to mitigate this by taking the microbiopsies from distinct points at the periphery of the lesion at each time point. The protocol has been edited in table 1, Schedule of events and section 9.5.2 Clinical samples to reflect this change. We have now made reference to this potential limitation within the discussion section.  

 ------------------------------------------------------------------------------------------------------------------

 (III) **Reviewer Concern: **If the hypothesis proposed by the authors cannot be met in Phase A, Phase B will need to be redesigned accordingly. The assertion made by the authors that minimally invasive skin biopsies will be at least 90% as effective as standard punch biopsies lacks empirical evidence or justification. Authors should propose an alternative plan if Phase A cannot meet the predefined targets. 

**Author Response: **We agree that further mitigation is required. We have submitted a substantial amendment and will now perform punch biopsies in Phase B of the study so that mycobacterial counts from serial minimally invasive skin sampling on days 2, 7 and 14 can be compared to culture results from punch biopsy on day 14. We have now edited the protocol in table 1, Schedule of events and section 9.5.2 Clinical samples to reflect this change.  

 ------------------------------------------------------------------------------------------------------------------

 (IV) **Reviewer Concern: **Given that prior exposure to environmental mycobacteria can influence the outcome of CHIM through cross-reactive immune responses, tuberculin (PPD) skin positivity should be regarded as one of the exclusion criteria. Cross-reactive immune responses can potentially complicate the assessment of BCG recovery. This altered baseline immune status might affect the kinetics and magnitude of immune responses and clearance of BCG. Including participants with pre-existing immune responses to environmental bacteria could significantly impact the interpretation of study outcomes.

**Author Response: **We are conducting our study in parallel with a BCG CHIM in Malawi. Given the majority of the population in Malawi are vaccinated with BCG we would not want to exclude those with TST reactivity as false positives can result from either BCG vaccination post infancy (10) or exposure to non-tuberculous mycobacterium (NTM) (11).

 Furthermore, given we would want this model to be representative of the population in a high TB burden setting, future inclusion of participants who may have sensitisation to NTM is important. BCG has been shown to be less effective in regions closer to the equator and it is thought that prior NTM exposure may be relevant(12) hence the importance of including this population in a TB CHIM. Previous research has shown that false positive rates for TST in high burden settings due to NTM sensitisation are low (2.3% in India) and lower in low burden settings, so we do not foresee this should be a significant issue in the present study (10).

 However, we do acknowledge that prior NTM sensitisation may affect the BCG immune response, and hence we will consider CHIM inclusion of TST in the screening for the Malawi BCG CHIM which would facilitate subgroup analysis.  

 1. Gordon SB, Sichone S, Chirwa AE, Hazenberg P, Kafuko Z, Ferreira DM, et al. Practical considerations for a TB controlled human infection model (TB-CHIM); the case for TB-CHIM in Africa, a systematic review of the literature and report of 2 workshop discussions in UK and Malawi. Wellcome Open Res. 2023;8:71.

 2. Zelmer A, Carroll P, Andreu N, Hagens K, Mahlo J, Redinger N, et al. A new in vivo model to test anti-tuberculosis drugs using fluorescence imaging. J Antimicrob Chemother. 2012;67(8):1948-60.

 3. Sabine Ehrt SF. Conditionally replicating Mycobacterium tuberculosis vaccines

 4. Mangtani P, Abubakar I, Ariti C, Beynon R, Pimpin L, Fine PE, et al. Protection by BCG vaccine against tuberculosis: a systematic review of randomized controlled trials. Clin Infect Dis. 2014;58(4):470-80.

 5. Jarvela J, Moyer M, Leahy P, Bonfield T, Fletcher D, Mkono WN, et al. Mycobacterium tuberculosis-Induced Bronchoalveolar Lavage Gene Expression Signature in Latent Tuberculosis Infection Is Dominated by Pleiotropic Effects of CD4(+) T Cell-Dependent IFN-γ Production despite the Presence of Polyfunctional T Cells within the Airways. J Immunol. 2019;203(8):2194-209.

 6. Bell LC, Pollara G, Pascoe M, Tomlinson GS, Lehloenya RJ, Roe J, et al. In Vivo Molecular Dissection of the Effects of HIV-1 in Active Tuberculosis. PLoS Pathog. 2016;12(3):e1005469.

 7. Tabone O, Verma R, Singhania A, Chakravarty P, Branchett WJ, Graham CM, et al. Blood transcriptomics reveal the evolution and resolution of the immune response in tuberculosis. J Exp Med. 2021;218(10).

 8. Harris SA, Meyer J, Satti I, Marsay L, Poulton ID, Tanner R, et al. Evaluation of a Human BCG Challenge Model to Assess Antimycobacterial Immunity Induced by BCG and a Candidate Tuberculosis Vaccine, MVA85A, Alone and in Combination. Journal of Infectious Diseases. 2013;209(8):1259-68.

 9. Davids M, Pooran A, Hermann C, Mottay L, Thompson F, Cardenas J, et al. A Human Lung Challenge Model to Evaluate the Safety and Immunogenicity of PPD and Live Bacillus Calmette-Guérin. Am J Respir Crit Care Med. 2020;201(10):1277-91.

 10. Farhat M, Greenaway C, Pai M, Menzies D. False-positive tuberculin skin tests: what is the absolute effect of BCG and non-tuberculous mycobacteria? Int J Tuberc Lung Dis. 2006;10(11):1192-204.

 11. Detjen AK, Keil T, Roll S, Hauer B, Mauch H, Wahn U, et al. Interferon-gamma release assays improve the diagnosis of tuberculosis and nontuberculous mycobacterial disease in children in a country with a low incidence of tuberculosis. Clin Infect Dis. 2007;45(3):322-8.

 12. Mangtani P, Abubakar I, Ariti C, Beynon R, Pimpin L, Fine PEM, et al. Protection by BCG Vaccine Against Tuberculosis: A Systematic Review of Randomized Controlled Trials. Clinical Infectious Diseases. 2013;58(4):470-80.

# Reviewer report: Reviewer response for version 1

**DOI:** 10.21956/wellcomeopenres.21943.r68106

**Published:** 2023-10-23

**Authors:** Valerie Koeken, Todia Setiabudiawan

**Affiliations:** 1 Radboud University Medical Center, Nijmegen, The Netherlands; 2 Research Centre Innovations in Care, Rotterdam University of Applied Sciences, Rotterdam, The Netherlands

**Overview and general comments**

 This two-phase design study protocol aims to refine the existing BCG-controlled human infection model (BCG-CHIM) as a surrogate for tuberculosis-controlled human infection model (TB-CHIM). In the first-phase, the objective is to test the safety of the BCG challenge and compare the gold standard punch biopsy with minimally invasive method to quantify BCG at the challenge site. Using the minimally invasive method selected from the first phase, it will enable longitudinal quantification of BCG at the challenge site for five time points over a 28-day follow-up period for the second phase. Also, longitudinal sampling of skin, blood, and respiratory mucosa will enable investigation of the immune response. The protocol is very clear and well-put, but there are some concerns that the authors should address.

**1. BCG-CHIM**

 Using BCG-CHIM for vaccine or therapeutic testing could primarily indicate immune protection against BCG rather than *Mtb*. BCG lacks crucial *Mtb* elements like the RD1 region which consist ESAT6/CFP10 and another antigen such as Ag85B, which BCG does produce but not secrete. Why not consider MTBVAC, a potentially more accurate challenge agent for *Mtb*? Lastly, for clarity and future applicability, could you elaborate on how the study might adapt to incorporate alternative TB challenge agents as they become available?

 Phase A is as a safety pilot for high-dose (4x) adult BCG vaccination, while Phase B explores high-dose BCG effects over time. Both are very interesting, but need clarification about their relevance to *Mtb* infection and how much the immune response against high-dose BCG challenge could mimic *Mtb *infection.

**2.** **BCG-Vaccinated participants**

 As the study is pilot in nature, I think it is better to also include people with a history of BCG vaccination, especially considering the future plans to extend this to Malawi. Adding individuals with a history of BCG vaccination might give early, valuable insights for TB-endemic regions. This way, at least you can be sure that BCG-CHIM is also safe in BCG-vaccinated adults, which most people in TB-endemic regions are. 

**3. Nasal sampling**

 Using nasal samples to measure respiratory mucosa immune response is very interesting and exciting, but it's usability remains poorly understood. How well do these samples correlate with immune responses in the lower respiratory tract where *Mtb* resides?

Is the study design appropriate for the research question?

Yes

Is the rationale for, and objectives of, the study clearly described?

Partly

Are sufficient details of the methods provided to allow replication by others?

Yes

Are the datasets clearly presented in a useable and accessible format?

Not applicable

Reviewer Expertise:

Innate immunity, trained immunity, BCG vaccination, Mtb infection, immunometabolism, multi-omics, tuberculosis.

We confirm that we have read this submission and believe that we have an appropriate level of expertise to confirm that it is of an acceptable scientific standard, however we have significant reservations, as outlined above.

# Response

**Published:** 2024-04-29

**Authors:** Emma Carter

**Affiliations:** Department of Clinical Sciences, Liverpool School of Tropical Medicine, Liverpool, UK

Responses to reviewer 1 comments-

 (I) **Reviewer Concern:** Using BCG-CHIM for vaccine or therapeutic testing could primarily indicate immune protection against BCG rather than Mtb. BCG lacks crucial Mtb elements like the RD1 region which consist ESAT6/CFP10 and another antigen such as Ag85B, which BCG does produce but not secrete. Why not consider MTBVAC, a potentially more accurate challenge agent for Mtb? Lastly, for clarity and future applicability, could you elaborate on how the study might adapt to incorporate alternative TB challenge agents as they become available?

**Author Response: **Thank you for these suggestions. We have made the choice to select BCG as our initial challenge agent to optimise the TB CHIM for several reasons, although we acknowledge the limitations of this as a challenge model for pulmonary tuberculosis. A workshop discussion taking place in Liverpool and Malawi agreed that BCG was currently the preferred agent to optimise a TB CHIM as it is licensed in both countries, has a long-established safety profile, has been used as control agent in novel vaccine studies, and has been reliably recovered from previous challenge models(1). 

 When alternative challenge agents are considered, including genetically modified *Mycobacterium tuberculosis, *a detailed assessment of transmission risk and government regulatory approval would be required as well as dose optimisation for optimal mycobacterial recovery. These considerations require significant investment and time, and whilst these are underway, we have made a decision to commence with BCG as a pilot agent. Regarding the use of MTBVac as a human challenge agent, we agree that this may have potential as a future challenge agent but would also require regulatory approval for use. When incorporating new challenge agents into the model, we plan to use techniques we have optimised for microsampling, mycobacterial recovery including culture and PCR techniques as well as single cell analysis and spatial transcriptomics of the immune response specific to mycobacteria *. *The BCG CHIM allows us to safely develop these. In terms of specific alternative challenge agents, BCG strains are under development that involve fluorophore insertion which would allow in vivo serial monitoring of BCG growth without the need for tissue sampling (2).

 Incorporation of this into a CHIM would require comparison of the fluorescent signal with serial tissue sampling used in our current model to ensure concordance with mycobacterial growth ex vivo. Conditionally replicating suicide strains of M.tb have been developed and are undergoing further testing in animals to ensure safety (3).

 They would would then need first-in-to-human studies to assure suicide on removal of the conditioning agent prior to incorporation into CHIMs.Further explanation of the rationale for selection of BCG as a challenge agent has been added to the introduction and discussion. Additional information has been added to the discussion in to elaborate on how future challenge agents may be incorporated into the model. 1b.

 Phase A is as a safety pilot for high-dose (4x) adult BCG vaccination, while Phase B explores high-dose BCG effects over time. Both are very interesting, but need clarification about their relevance to Mtb infection and how much the immune response against high-dose BCG challenge could mimic Mtb infection. A recently conducted systematic review and workshop in Liverpool and Malawi highlighted the limitations of an intradermal BCG CHIM and the extent to which it can be used to model M.tb infection in TB vaccine evaluation. Firstly, the immune response to WT- *M.tb *is complex and can lead to a wide range of disease manifestations after initial primary infection. Furthermore, the immune response to BCG is variable according to age, geography and prior non-tuberculous Mycobacterium exposure and the correlates of protection are not fully known(4).

 Finally, the compartmental skin response to intradermal BCG will likely differ to pulmonary *M.tb* given the variation in immune populations at these sites (5).

 However, one study has shown concordance between the transcriptional profile of skin punch biopsy specimens from TST injection sites with human pulmonary granulomas suggesting correlation between the compartmental skin and pulmonary immune response to *M.tb* *(6)* *.*

 This study aims to provide a detailed analysis of the immune response over time to BCG in skin, respiratory mucosa and systemically which has not previously been conducted and could be compared with emerging work analysing the host transcriptional response to WT- *M.tb* exposure (7).

 Further detail has been added to the discussion in response to this comment.

 ------------------------------------------------------------------------------------------------------------------

 (II) **Reviewer Concern:** As the study is pilot in nature, I think it is better to also include people with a history of BCG vaccination, especially considering the future plans to extend this to Malawi. Adding individuals with a history of BCG vaccination might give early, valuable insights for TB-endemic regions. This way, at least you can be sure that BCG-CHIM is also safe in BCG-vaccinated adults, which most people in TB-endemic regions are.

**Author Response: **Many thanks for this valuable comment. We agree that it will be important to include people with a history of prior BCG vaccination. Of note, BCG CHIM has previously been safely undertaken in Oxford in participants with prior BCG vaccination(8). After completion of this pilot study, we plan to expand to include previously BCG vaccinated individuals and compare the immune response and mycobacterial recovery to those that have not previously received BCG. We have now added to the discussion explaining that this model in Malawi will include previously BCG vaccinated individuals.

 ------------------------------------------------------------------------------------------------------------------

 (III) **Reviewer Concern: **Using nasal samples to measure respiratory mucosa immune response is very interesting and exciting, but it's usability remains poorly understood. How well do these samples correlate with immune responses in the lower respiratory tract where Mtb resides?

**Author Response: **We agree that at present the correlation between nasal mucosa and lower respiratory tract mucosal immune responses to Mtb is not yet known. Our results of nasal mucosal immune responses can be compared with recently published literature describing the immune response to BCG in lower respiratory tract samples (9).

 Although it is beyond the scope of this current protocol, we plan on undertaking bronchoscopy in future work to compare nasal mucosal samples to lower respiratory samples in BCG CHIM participants (9).

 1. Gordon SB, Sichone S, Chirwa AE, Hazenberg P, Kafuko Z, Ferreira DM, et al. Practical considerations for a TB controlled human infection model (TB-CHIM); the case for TB-CHIM in Africa, a systematic review of the literature and report of 2 workshop discussions in UK and Malawi. Wellcome Open Res. 2023;8:71.

 2. Zelmer A, Carroll P, Andreu N, Hagens K, Mahlo J, Redinger N, et al. A new in vivo model to test anti-tuberculosis drugs using fluorescence imaging. J Antimicrob Chemother. 2012;67(8):1948-60.

 3. Sabine Ehrt SF. Conditionally replicating Mycobacterium tuberculosis vaccines

 4. Mangtani P, Abubakar I, Ariti C, Beynon R, Pimpin L, Fine PE, et al. Protection by BCG vaccine against tuberculosis: a systematic review of randomized controlled trials. Clin Infect Dis. 2014;58(4):470-80.

 5.Jarvela J, Moyer M, Leahy P, Bonfield T, Fletcher D, Mkono WN, et al. Mycobacterium tuberculosis-Induced Bronchoalveolar Lavage Gene Expression Signature in Latent Tuberculosis Infection Is Dominated by Pleiotropic Effects of CD4(+) T Cell-Dependent IFN-γ Production despite the Presence of Polyfunctional T Cells within the Airways. J Immunol. 2019;203(8):2194-209.

 6. Bell LC, Pollara G, Pascoe M, Tomlinson GS, Lehloenya RJ, Roe J, et al. In Vivo Molecular Dissection of the Effects of HIV-1 in Active Tuberculosis. PLoS Pathog. 2016;12(3):e1005469.

 7.Tabone O, Verma R, Singhania A, Chakravarty P, Branchett WJ, Graham CM, et al. Blood transcriptomics reveal the evolution and resolution of the immune response in tuberculosis. J Exp Med. 2021;218(10).

 8. Harris SA, Meyer J, Satti I, Marsay L, Poulton ID, Tanner R, et al. Evaluation of a Human BCG Challenge Model to Assess Antimycobacterial Immunity Induced by BCG and a Candidate Tuberculosis Vaccine, MVA85A, Alone and in Combination. Journal of Infectious Diseases. 2013;209(8):1259-68.

 9. Davids M, Pooran A, Hermann C, Mottay L, Thompson F, Cardenas J, et al. A Human Lung Challenge Model to Evaluate the Safety and Immunogenicity of PPD and Live Bacillus Calmette-Guérin. Am J Respir Crit Care Med. 2020;201(10):1277-91.

 10. Farhat M, Greenaway C, Pai M, Menzies D. False-positive tuberculin skin tests: what is the absolute effect of BCG and non-tuberculous mycobacteria? Int J Tuberc Lung Dis. 2006;10(11):1192-204.

 11. Detjen AK, Keil T, Roll S, Hauer B, Mauch H, Wahn U, et al. Interferon-gamma release assays improve the diagnosis of tuberculosis and nontuberculous mycobacterial disease in children in a country with a low incidence of tuberculosis. Clin Infect Dis. 2007;45(3):322-8.

 12. Mangtani P, Abubakar I, Ariti C, Beynon R, Pimpin L, Fine PEM, et al. Protection by BCG Vaccine Against Tuberculosis: A Systematic Review of Randomized Controlled Trials. Clinical Infectious Diseases. 2013;58(4):470-80.
